# Commencements in N. Y. Medical Schools

**Published:** 1860-04

**Authors:** 


— Since our last issue, the Medical Schools in this city have all closed their winter sessions. The New York Medical College held its Eighth Annual Commencement on Thursday evening, March 1. The regular degree of Doctor in Medicine was conferred on *twenty* candidates in course, and the honorary degree on Samuel T. Parker and Campbell Morfitt, of New York; Thomas Garrett, of Pennsylvania; and Rev. A. G. Shears, of Conn. Samuel J. Tilden, Esq., delivered the Valedictory Address.

The *New York University, Medical Department*, held its Commencement on Wednesday evening, March 7. There were *one hundred and thirty-eight* graduates. Prof. Valentine Mott gave the Valedictory Address.

At the Commencement of the College of Physicians and Surgeons, which was held the evening of March 8, there were *fifty-five* graduates. Dr. Seth Lyman Chase, one of the graduates, delivered the Valedictory to the Class, and Prof. T. W. Markoe delivered the Address to the Alumni.

